# Effects of heparin on the uptake of lipoprotein lipase in rat liver

**DOI:** 10.1186/1472-6793-4-13

**Published:** 2004-11-15

**Authors:** Lucyna Neuger, Senén Vilaró, Carmen Lopez-Iglesias, Jitendra Gupta, Thomas Olivecrona, Gunilla Olivecrona

**Affiliations:** 1 Department of Surgical and Perioperative Sciences, Clinical Physiology, Umeå University, Umeå, Sweden; 2 Department of Cell Biology, University of Barcelona, Barcelona, Spain; 3 Scientific and Technical Services, University of Barcelona, Barcelona, Spain; 4 Department of Medical Biosciences, Physiological Chemistry, Umeå University, Umeå, Sweden

## Abstract

**Background:**

Lipoprotein lipase (LPL) is anchored at the vascular endothelium through interaction with heparan sulfate. It is not known how this enzyme is turned over but it has been suggested that it is slowly released into blood and then taken up and degraded in the liver. Heparin releases the enzyme into the circulating blood. Several lines of evidence indicate that this leads to accelerated flux of LPL to the liver and a temporary depletion of the enzyme in peripheral tissues.

**Results:**

Rat livers were found to contain substantial amounts of LPL, most of which was catalytically inactive. After injection of heparin, LPL mass in liver increased for at least an hour. LPL activity also increased, but not in proportion to mass, indicating that the lipase soon lost its activity after being bound/taken up in the liver. To further study the uptake, bovine LPL was labeled with ^125^I and injected. Already two min after injection about 33 % of the injected lipase was in the liver where it initially located along sinusoids. With time the immunostaining shifted to the hepatocytes, became granular and then faded, indicating internalization and degradation. When heparin was injected before the lipase, the initial immunostaining along sinusoids was weaker, whereas staining over Kupffer cells was enhanced. When the lipase was converted to inactive before injection, the fraction taken up in the liver increased and the lipase located mainly to the Kupffer cells.

**Conclusions:**

This study shows that there are heparin-insensitive binding sites for LPL on both hepatocytes and Kupffer cells. The latter may be the same sites as those that mediate uptake of inactive LPL. The results support the hypothesis that turnover of endothelial LPL occurs in part by transport to and degradation in the liver, and that this transport is accelerated after injection of heparin.

## Background

Lipoprotein lipase (LPL) hydrolyses triglycerides in chylomicrons and VLDL and thereby makes fatty acids available for cellular uptake and use in metabolic processes [1,2]. Relatively high levels of LPL mRNA are found in adipose tissue, heart, red skeletal muscle and lactating mammary gland [3,4]. Parenchymal cells, such as adipocytes and myocytes, synthesize and secrete the enzyme, which is then transferred to the endothelium and anchored to the oligosaccharide chains of heparan sulfate proteoglycans (HSPG) [1,2]. There is continuous recycling of the enzyme between the luminal and abluminal side of the endothelial cells, and perhaps to other extracellular sites in the tissue [1,5,6]. It is not known how the extracellular enzyme is turned over. One possibility is that it is transported with blood to the liver and degraded there [7]. LPL activity in the circulating blood is normally low and most of the LPL protein in blood is catalytically inactive [7-10]. Release of lipase from extrahepatic tissues into blood has been demonstrated [11,12]. Model studies with labeled LPL have demonstrated uptake and degradation of both active and inactive LPL in the liver [13-15].

Heparin releases LPL from its endothelial binding sites into the circulating blood. The uptake in the liver is retarded, but not abolished [13,14]. This has been taken as evidence that there are both heparin-sensitive and heparin-insensitive binding sites in the liver. An implication is that the high lipase activity in blood after heparin injection is due to release from peripheral tissues combined with retarded uptake in the liver. Studies in rats and in human subjects indicate that the net effect of heparin is an accelerated transport of LPL to the liver [16,17]. If this hypothesis is correct, LPL mass and activity should increase in the liver after injection of heparin, in contrast to the decrease that occurs in extrahepatic tissues [6]. To test these concepts we have followed LPL activity and mass in liver after injection of heparin, and we have used immunofluorescence to explore if heparin changes the pattern of where in the liver LPL binds.

## Results

### Amount and distribution of LPL in liver

LPL activity in rat liver was 26 ± 1 mU/g (Table 1), similar to the activity reported by Peterson et al [15]. This is low compared to the activities in adipose tissue (around 1600 mU/g in fed rats [6]) and heart (around 1100 mU/g [18]). LPL mass was 120 ng/g. The relation between LPL activity and mass in plasma was similar to that in liver; activity was 8 mU/ml and mass was 29 ng/ml (Table 1). The specific activity of the enzyme in plasma increased to around 1.2 after injection of heparin. This indicates that most of the LPL in plasma or liver before heparin was inactive, in accord with studies on LPL in human plasma [7-10].

**Figure 1:**
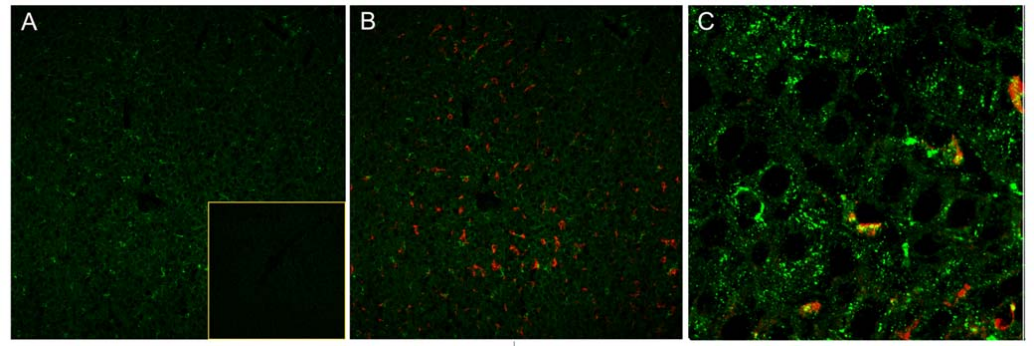
**Detection of endogenous LPL in rat liver by immunofluorescence**. Tissue sections were double-stained with chicken anti-LPL IgG (detected with Alexa 488-labeled goat anti-chicken antibodies (green)), and the monoclonal anti-Kupffer cell antibody ED2 (detected with Alexa 546-labeled goat anti-mouse antibodies (red)). All panels show sections from livers of rats that did not receive heparin. The magnification in panels A and B was × 20-fold whereas it was × 60 + zooming in panel C. Panel A shows staining only for LPL. Panel B is the same area as in A but with staining also for ED2. Panel C shows both stainings. The inset in panel A shows a control section with non-immune chicken IgG instead of anti-LPL.

To study the distribution of endogenous LPL in rat liver we used affinity-purified chicken antibodies raised against bovine LPL. These antibodies have previously been used for ELISA of LPL in rat tissues [19]. There was faint immunofluorescence in a granular pattern (green) over hepatocytes, and stronger staining over scattered cells (Figure 1). Some of these reacted positively with the ED2 antibodies indicating that they were Kupffer cells. Sections treated with pre-immune IgG instead of anti-LPL (inset in Fig 1), as well as sections where the second antibody was omitted, showed no immunofluorescence.

### Effects of heparin

LPL activity and mass in plasma increased many-fold after heparin injection (Table 1). The highest value was at 15 min, but even after 60 min the activity was still more then 50-fold higher than in normal plasma. In liver the level of LPL activity had increased already two min after heparin injection and it was about 10-fold increased both at 15 and 60 min compared to time 0. The amount of LPL protein in the liver, measured by the ELISA, had increased about two-fold at two min, 3.5-fold at 15 min and five-fold after 60 min. These data give direct evidence that after injection of heparin some of the LPL released into plasma was taken up by the liver.

Hepatic lipase (HL) was also measured in the livers (Table 1). As expected, but in sharp contrast to what was found for LPL, the HL activity decreased after heparin. Already after two min the activity had decreased by two thirds. This reflects the release of HL into the circulating blood. The activity in liver remained constant at 15 min and had begun to increase again after 60 min.

The pattern of immunofluorescence for LPL after heparin was similar to that before heparin, with faint staining over most cells and more intense staining over scattered cells, some of which were ED2 positive (not shown).

To explore the origin of the LPL released into plasma and taken up by the liver we measured LPL activity in heart and adipose tissue before and 20 min after injection of heparin (Table 2). Data for plasma and liver in fed rats were similar to those in the experiment in Table 1. In fasted rats, plasma post-heparin LPL activity was less than half of that in fed rats in accord with previous studies [18,20]. The LPL activity in liver increased after heparin, in concert with the results in Table 1. There was no statistically significant difference of the LPL activity in liver between fed and fasted rats either before or after heparin. In adipose tissue the LPL activity was about 5-fold higher in fed compared to fasted rats. Heparin caused a washout of 45 % of the LPL activity from epididymal and 65% from perirenal adipose tissue (p < 0.01). Values for LPL activity in heart were somewhat higher in fasted than in fed rats, but this did not reach statistical significance (p = 0.16). Heparin caused no significant decrease of heart LPL in fed rats, but a highly significant (p < 0.01) washout in the fasted rats, about 40%.

### Injection of labelled bovine LPL

The immunostaining of endogeneous LPL was faint and not suitable for detailed analysis or quantitation. The reason is that the only reagents available were chicken antibodies raised against bovine LPL. To further explore the hepatic binding and uptake of the enzyme we therefore injected bovine LPL. A trace amount of ^125^I-labeled LPL was included so that we could quantitate the uptake/metabolism. Values are given in Table 3 for the times at which localization of the lipase was studied by immunofluorescence. These values agree with an earlier study when more complete time curves were obtained [13]. For active LPL earlier studies have shown that at short times after injection about half of the lipase locates in extrahepatic tissues and about half in the liver [13,21,22]. In the present study 33 % of the radioactivity was in liver after two min (Table 3). This increased to 52 % after 15 min and then decreased again to 20 % after 60 min. Heparin slows down the clearance of LPL from the blood [13,21]. Fifteen min after injection of the labeled lipase, about half is still in the circulating blood [13]. In our experiments 30 % was in the liver at this time (Table 3). Hence, 60 % or more of the removal from plasma had taken place in the liver. After 60 min radioactivity in the liver had decreased to 23 % of the injected dose (Table 3). Earlier studies have demonstrated that acid soluble breakdown products of the labeled lipase appear in blood [13] and in the perfusion fluid in experiments with isolated livers [14]. Hence, the decrease of label in liver at longer times probably occurred through degradation of the lipase.

For inactive lipase earlier studies have shown that only a minor fraction locates in extrahepatic tissues, and more than 70% of the uptake occurs in the liver [13]. In the present study, 50 % of the radioactivity was in the liver 15 min after injection, while 9 % was in the blood. After 60 min the radioactivity in the liver had decreased to 19 % of the injected dose.

**Figure 2:**
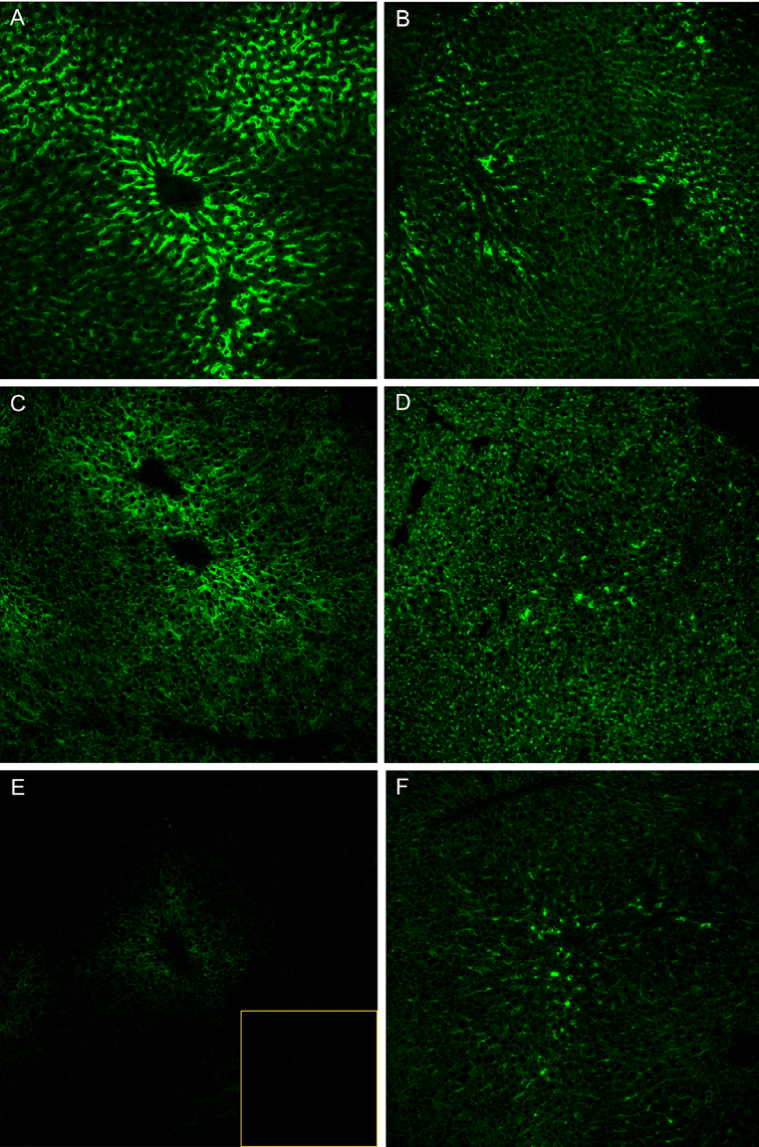
**Distribution of bovine LPL in livers at different times after injection, and the effect of heparin**. Tissue sections were stained with the rabbit polyclonal anti-LPL IgG and then with goat anti-rabbit IgG labelled with Alexa fluor 488 (green). Panels A, C and E are from rats that did not receive heparin. Panels B, D and F are from rats that had been given heparin five min before the injection of active bovine LPL. Panels A and B are two min, panels C and D are 15 min, and panels E and F are 60 min after injection of the lipase. The inset in panel E shows a section from the liver of a rat that was not injected with bovine LPL. The magnification was × 20.

To visualize the injected bovine LPL we used rabbit antibodies. These antibodies did not inhibit endogenous LPL in rat post-heparin plasma or in extracts of adipose tissue. No immuno-reaction was seen in sections from control rats not injected with LPL (inset in Figure 2E). Likewise, there was no immunofluorescence in sections treated with non-immune rabbit IgG instead of anti-LPL, or when the second antibody was omitted.

**Figure 3:**
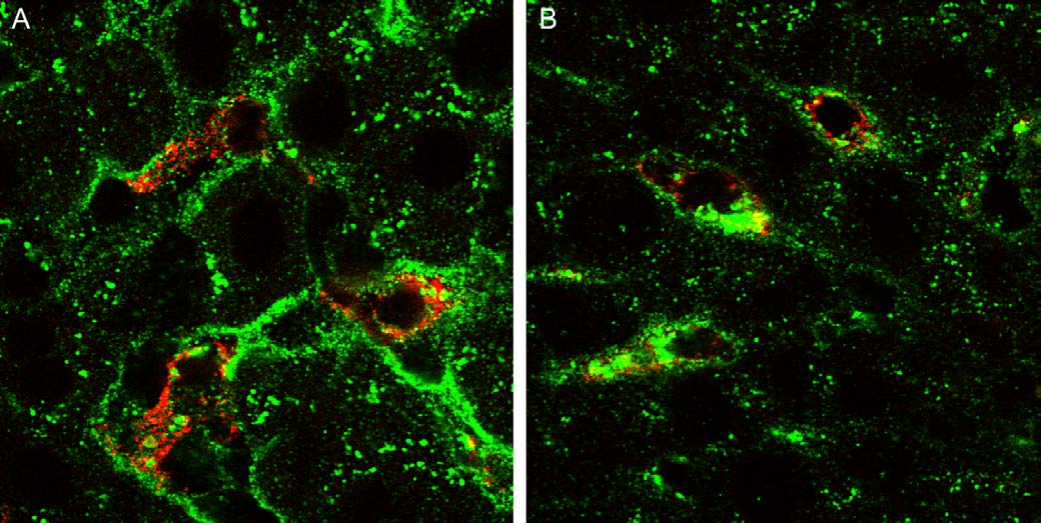
**Higher magnification of sections from the same experiment as in Figure 2 **Both sections are 15 min after injection of active bovine LPL. The rat in panel A did not receive heparin; the rat in panel B had been given heparin five min before LPL. Green colour represents staining of LPL by the rabbit polyclonal antibody. Red colour represents staining of Kupffer cells by the ED2 antibody. The magnification was × 60 + zooming.

Two min after injection of the active lipase, intense immuno-staining was seen along sinusoids (Figure 2A). This staining was strongest in the periportal areas. There was little staining outside the sinusoids. Occasionally a few fluorescent dots were seen in hepatocytes, possibly representing endocytic vesicles. At 15 min after injection there was still staining over the sinusoids (Figure 2C), but most of the staining was now associated with hepatocytes and the number of granulae seen in hepatocytes had increased, indicating that the lipase had been internalized in vesicles (Figure 3A). Some cells, localized predominantly around the portal area, had many fluorescent dots. This staining was mainly granular in contrast to the more continuous staining over sinusoids at this time. Double staining at 15 min after injection of LPL demonstrated that some of these cells were ED2-positive (Figure 3A). At 60 min little or no staining remained at sinusoidal surfaces and the total staining had decreased (Figure 2E), but there were still grains in cells close to the portal area. This was probably enzyme that had been taken up in intracellular vesicles and had not yet become degraded.

**Figure 4:**
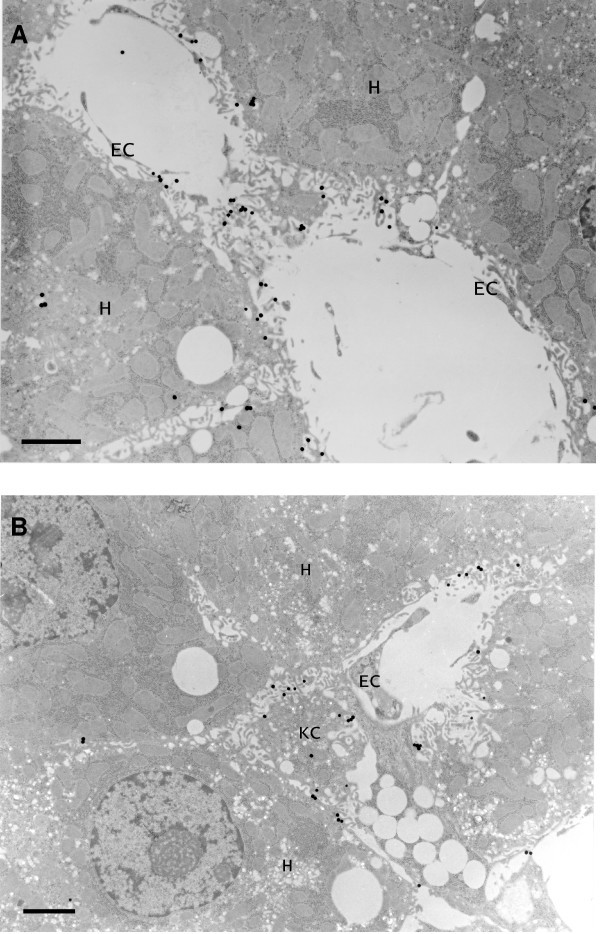
**Ultrastructural localization of injected bovine LPL **^125^I-labeled active (upper panel) or inactive (lower panel) LPL was injected. Ten min later the rats were killed and sections of their livers were processed for autoradiography as detailed in the methods section. H, hepatocytes, EC, endothelial cell, KC, Kupffer cell. Bar: 2 μm.

To get more detailed information about the binding sites in liver we used electron microscopy. For this, bovine ^125^I-labeled LPL was injected and 10 min later the livers were fixed by perfusion. The enzyme was visualized as silver grains by means of autoradiography (Figure 4A). Counting of silver grains in the sections showed that about 55% of the lipase was within spaces of Disse, mostly associated with hepatocytes. Twenty-five to 30% was on the luminal side of endothelial cells. Only about 15% was inside hepatocytes and other cell types, probably Kupffer cells.

**Figure 5:**
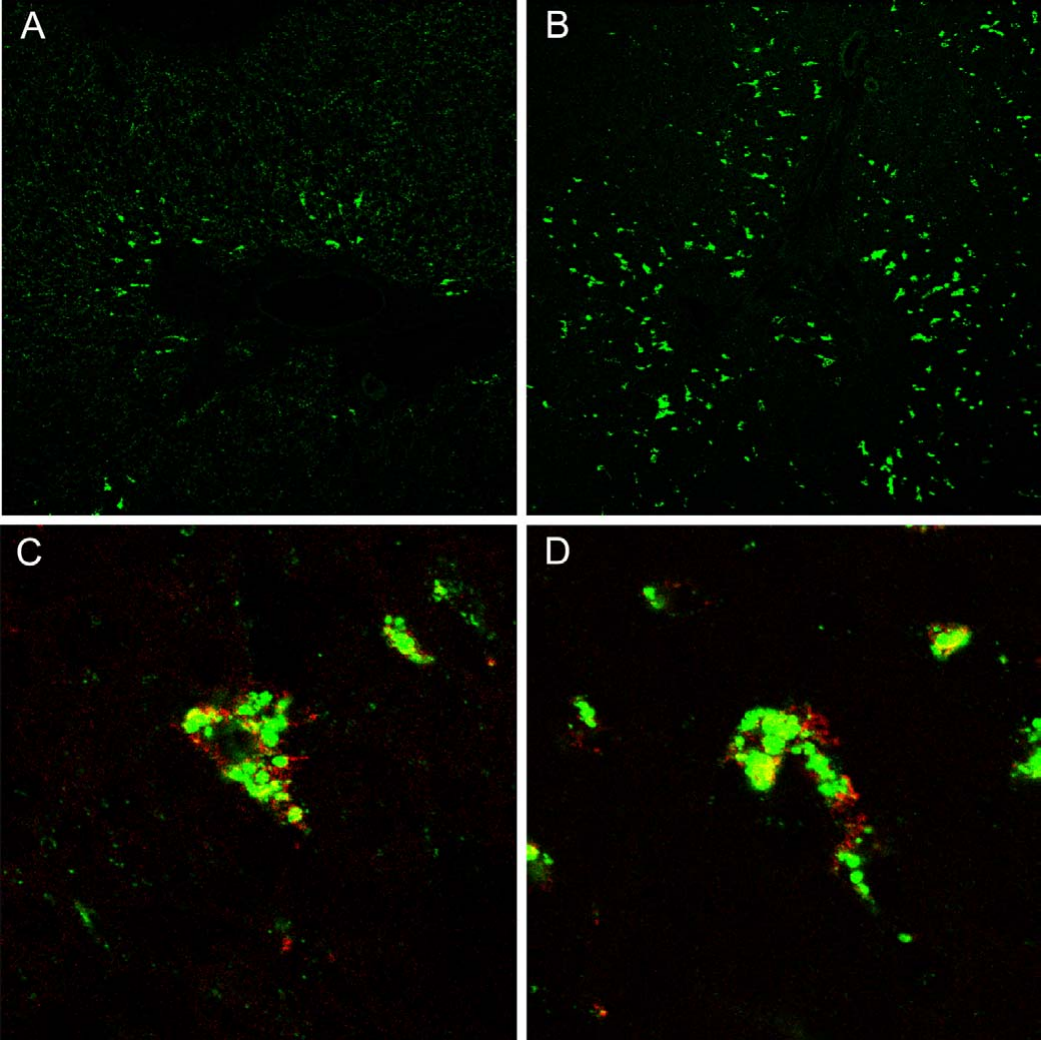
**Distribution of injected, inactive bovine LPL in livers, and the effect of heparin **All sections are 15 min after injection of inactive bovine LPL. The sections in panels A and C are from rats that did not receive heparin; the sections in panel B and D are from rats that had been given heparin five min before the lipase. Green colour represents staining of LPL by the rabbit polyclonal antibody. Red colour represents staining of Kupffer cells by the ED2 antibody. Panels C and D show both stainings. The magnification was × 20 in panels A and B; × 60 + zooming in panels C and D.

The pattern of distribution was quite different for the inactive lipase. At the first time, two min, there was intense staining for LPL over scattered cells concentrated to the portal regions but very little staining over sinusoids or hepatocytes (not shown). Double staining with the ED2 antibody showed that the intensively LPL-positive cells were Kupffer cells. After 15 min the immunofluorescence had changed to a more punctuate pattern that still co-localized with Kupffer cells (Figure 5C). This indicated that the lipase had been internalized in vesicles. There was some immunostaining over other cells, presumably hepatocytes, but this was much weaker than the staining over Kupffer cells (Figure 5A and 5C). After 60 min the intensity of the staining had decreased, but the pattern with more intense staining over Kupffer cells and much weaker staining over other cells remained (not shown). Hence, there was no indication that the inactive lipase first bound to one type of cell and then transferred to another type for uptake. Electron microscopic autoradiography of sections from livers of rats ten min after injection the inactive LPL, confirmed that the labelled lipase was mostly associated to sinusoidal cells that morphologically seemed to resemble Kupffer cells.

Heparin markedly changed the pattern of localization for the active lipase. The initial (two min after injection) staining along the sinusoids was much weaker than in sections from rats that had not received heparin. The staining was generally more intense at 15 min compared to at two min after injection of the lipase (Figure 2D). This is in accord with the radioactivity data that showed that more LPL had been taken up (Table 2). Compared to the pattern without heparin, the staining was spread throughout the liver parenchyma rather than concentrated in the portal areas (compare Figure 2A,2C and 2E with 2B,2D and 2F). There was more staining associated with scattered cells in the portal areas than in sections from rats that had not received heparin (Figure 2B). These cells were ED2-positive (not shown). Already at 15 min the LPL-staining had taken on a granular character both in the ED2-positive cells and in hepatocytes (Figure 3). At 60 min the intensity of staining had decreased (Figure 2F). The ED2 positive cells still dominated the picture but there was also granular staining over hepatocytes. More staining remained compared to the same time point without heparin (compare Figure 2F and 2E).

Electron microscopic autoradiography showed that when heparin was injected ten min after active LPL there was a strong reduction in the amount of LPL in the spaces of Disse and on endothelial cells, while the radioactivity found in hepatocytes and Kupffer cells remained (data not shown).

Heparin had no marked effect on the distribution of the inactive LPL (Figure 5). Most of the staining co-localized with staining for ED2 positive Kupffer cells (Figure 5D).

## Discussion

This study shows that after injection of heparin, LPL activity and mass in liver increases several-fold, in concert with the hypothesis that heparin causes accelerated transport of LPL to the liver. In other parts of the body LPL is attached to HSPG [1,2]. Heparin competes efficiently with these binding sites. The rapid extraction of LPL by the liver in the presence of heparin implies that some other type of binding site must be present there. Members of the LDL receptor (LDL-R) family bind both the active and the inactive form of the lipase [23] and two recent studies indicate that LRP is involved in hepatic uptake of LPL [24,25]. The binding of active LPL to LRP is, however, strongly impeded by heparin [26]. Therefore, it is unlikely that the heparin-resistant binding of active LPL is mediated by LRP or some other receptor of the LDL-R family.

Heparin markedly decreased the binding of LPL along the sinusoids. This presumably reflects that binding to HSPG was competed by heparin. Staining associated with Kupffer-like cells increased. This may be the same sites as those that bind inactive LPL. Another possibility is that the LPL-heparin complexes were recognized and taken up as such. There is evidence for binding and uptake of heparin by Kupffer cells [27]. Most of the binding was, however, to hepatocytes even in the presence of heparin. Our data are qualitative, based on the immunolocalization. For more accurate quantitation one should label the lipase with a non-degradable label like ^125^I-tyramine cellobiose and isolate the different cell types.

It has been suggested that LPL and HL bind, at least in part, to the same sites in the liver [24,28]. It is, however, unlikely that HL shares the heparin-insensitive sites. The response of the two enzymes to heparin was very different. HL activity decreased by 60% within two min after heparin injection, reflecting release of the lipase into blood. In contrast, LPL activity in the liver increased, reflecting binding to the heparin-insensitive sites.

Earlier studies have shown that there are also heparin-sensitive sites that bind LPL in liver [13]. Wallinder et al perfused livers in situ with heparin 15 min after injection of ^125^I-LPL to rats and found that about 10% of the lipase that had bound in the liver could be released [13]. Vilaró et al perfused isolated livers with ^125^I-LPL in a recirculating system for 10 min. After wash the perfusion was then continued in single pass mode with a heparin-containing medium. About 50% of the LPL that had bound in the liver reappeared in the perfusion medium within four min [14,29]. At least some of these heparin-sensitive sites are likely HSPG, and they are probably the main sites that mediate the initial capture of LPL from blood. This binding may correspond to the decoration of the sinusoids seen by immunofluorescence at the earliest time after injection of the lipase. HSPG are present on virtually all cells in the body, including hepatocytes and endothelial cells in the liver [30,31]. Vilaró et al studied binding and uptake of LPL in cultured hepatocytes [32]. The enzyme was concentrated at the tips of the microvilli, a site where also HSPG are highly abundant [33,34]. Immunofluorescence now showed that at short times after injection the lipase located along the sinusoids, and electron microscopy showed that most of the lipase was in the spaces of Disse. The immunofluorescence was strongest in the portal areas, indicating that the lipase was extracted soon after it entered the liver. Most of the staining was over hepatocytes. With time the immunofluorescence shifted to a granular pattern, indicating that the enzyme had been internalized. The internalization may have occurred with lipase bound to HSPG, as demonstrated with cultured fibroblasts and with hepatocytes [32,35,36] and/or with lipase bound to LRP as suggested by several authors [24,25,35,37]. In fibroblasts, both these pathways contribute to LPL internalization [38]. The lipase may well recycle as demonstrated by Heeren et al in experiments with cultured hepatocytes [39,40]. The immunostaining gradually faded with time indicating that the lipase was degraded, in accord with previous studies, and with the decrease of ^125^I-radioactivity in the liver observed in the present study.

Inactive LPL, as prepared here, was taken up in Kupffer cells. Most of the LPL in plasma is inactive [8,10] and there is inactive LPL also in the tissues [19]. The nature and metabolic significance of this inactive LPL is not clear. Western blot analysis indicates that it is full-length LPL [7]. On heparin-agarose it elutes in the position expected for monomeric LPL [7]. Gel filtration of plasma indicates that it is associated with the lipoproteins [8]. The turnover of inactive LPL does not appear to be much influenced by heparin [6,8,13]. Earlier studies had shown that injected, inactive LPL is rapidly bound and degraded in the liver, both *in vivo *[13] and on perfusion through an isolated liver [14]. In these studies the inactive lipase was produced by complete denaturation in 6 M guanidinium chloride. In the present study the inactive lipase was gently prepared by incubation in rat plasma at 45°C. This probably results in dissociation to inactive but still folded monomers [41,42]. Our preliminary experiments had shown that under these conditions the enzyme slowly lost its catalytic activity. After 90 min, the time used here, less than 5 % of the activity remained. In terms of clearance rate and tissue distribution, the present preparation behaved as the fully denatured lipase used in previous studies. Whether any of these lipase preparations faithfully reproduces the metabolic behaviour of the inactive lipase present in plasma is not clear. A recent study has defined several conformational states of the LPL molecule, and the kinetics of conformational transitions [42].

Before heparin, the ratio of LPL activity to LPL mass was about 0.2 mU/ng in liver and 0.3 mU/ng in blood (Table 1). After heparin, the specific activity for LPL in plasma increased to about 1.2, indicating that heparin released mainly or almost exclusively the active form of the lipase as has been shown to be the case in humans [7]. In the liver, however, the ratio stayed well below one. At the 60 min time point LPL mass had increased by about 500 ng/g, but LPL activity had only increased by about 240 mU. This indicates that the lipase loses catalytic activity after it is taken up in the liver, but is degraded more slowly. These observations are in accord with earlier studies. Chajek-Shaul et al. perfused rat livers with LPL-containing media and found that the enzyme lost its catalytic activity soon after binding/uptake in liver [43]. Wallinder et al compared the uptake and degradation of ^125^I-labeled LPL in liver to that for asialofetuin, which is taken up by the galactose receptor [13]. The half-life for asialofetuin was about 15 min, whereas that for the lipase was longer, about one hour.

To explore the source of LPL released into plasma and taken up by the liver we measured LPL activity in adipose tissue and heart before and after injection of heparin. In fed rats there was a large decrease of LPL activity in adipose tissue, in accord with a previous study [6]. From these data and the tissue weights at least 7000 mU LPL activity was washed out from white adipose tissue during the first 20 min after heparin. To this should be added an unknown amount of LPL washed out from other tissues. During the same time the LPL activity increased by the 1600 mU in the liver and about 5100 mU in blood. These data indicate that the dominant source of LPL released to plasma in fed rats is the white adipose tissue. Post-heparin LPL activity was lower in fasted than in fed rats, less than half, in accord with previous studies [18,20]. LPL activity in adipose tissue is suppressed during fasting, and we did not find any significant loss of activity after heparin. Hence, the adipose tissue releases much less LPL activity into plasma in fasted than in fed rats. The main contributors are presumably heart and skeletal muscle. We observed a large washout of LPL activity from heart in the fasted rats, about 40%. In other studies we have noted a similar washout from the *Soleus *muscle. Kuwajima et al perfused some of the rat hindlimb muscles (gastrocnemius, soleus and plantaris) with heparin in situ and observed a large release in fasted but not in fed rats [20]. These data suggest that in fasted rats, the main source of LPL released into plasma by heparin are skeletal muscles and heart.

LPL in plasma is bound to lipoproteins and it has been suggested that the lipase serves as a ligand for binding and uptake of lipoproteins in the liver. Chevreuil et al injected doubly labelled chylomicrons to rats shortly after heparin [44]. The results showed accelerated lipolysis of the triglyceride moiety of the chylomicrons, as expected. In addition, clearance of chylomicron remnants, as traced by retinyl esters, was greatly accelerated. Together with the present results this suggests that after heparin, the large increase of LPL in blood may accelerate the hepatic uptake of some lipoproteins.

## Conclusions

• In the liver, the active form of LPL initially binds to sinusoidal surfaces but then transfers to and is taken up mainly in hepatocytes

• An inactive form of LPL, presumably monomers, was mainly taken up in Kupffer cells

• Heparin retards the uptake of active LPL in liver, but there are heparin insensitive binding sites for LPL both on hepatocytes and on Kupffer cells

• Release of LPL into blood by heparin results in accelerated transport of the lipase to the liver

• The observation that rat liver contained substantial amounts of LPL, most of which was inactive, is in accord with the hypothesis that one route for turnover for endothelial LPL is transport to and degradation in the liver

• The observations that most of the LPL in blood is inactive, that injected inactive bovine LPL located to Kupffer cells, and that the immunostaining for endogenous LPL was more intense over Kupffer cells than over hepatocytes suggest that a substantial fraction of the transport from extrahepatic tissues occurs with LPL that has lost its activity.

• The main source of LPL released into plasma and taken up by the liver in fed rats is the adipose tissue, whereas in fasted rats the main sources are heart and skeletal muscles.

## Methods

### Animals

Male Sprague-Dawley rats (Moellegard Breeding centre, Denmark) weighing 180–220 g were used. They were kept on a standard pellet diet in a 12-hour light cycle. In order not to disturb blood circulation or the metabolic functions of the liver, we performed all experiments on unanaesthetized rats. They were killed through decapitation at the time of tissue removal. Injections were made in the tail vein. Mean liver weight for the rats was about 9 g. In some of the rats we dissected out all visible adipose tissue. The mean total weight was 14 g, including fibrous tissue removed with the subcutaneous adipose tissue. To correct values for LPL mass/activity in the liver we used an estimated figure of 3% for the amount of blood plasma remaining in the liver after exsanguination. This was based on earlier experiments with ^125^I-albumin and Cr^51^-labeled red blood cells [45,46]. The local Animals Care Committee in Umeå approved all animal procedures.

### Materials

Vectashield mounting medium was from Vector Laboratories, Burlingame, CA. Tissue-Tec OCT compound was purchased from Sakura Finetek Europe BV, Zoeterwoude, The Netherlands. Microscope slides and cover slips were from Menzel – Gläser, Germany. Plasma, used in the preparation of catalytically inactive LPL, was taken from fasted rats with EDTA as anticoagulant. Heparin was obtained from Leo Pharma AB, Malmö, Sweden. The dose given was 500 IU/kg body weight.

### Lipase and antibody preparations

LPL was purified from bovine milk as previously described [47] and was labeled with ^125^I using the lactoperoxidase/glucose oxidase method [13]. The labeled LPL was separated from damaged protein and free iodine by chromatography on heparin-Sepharose using a gradient of NaCl. The labeled preparations were stored at -70°C in the presence of 2 mg BSA per ml. The specific activity of the labeled LPL was approximately 10 000 cpm/ng. Inactive LPL for the electron microscopy study was prepared by dissociation in guanidinium hydrochloride as described [13]. To find suitable conditions to prepare inactive LPL for the immunofluorescence experiments, we diluted bovine LPL in rat plasma to the concentration we would later use in the *in vivo *experiments and incubated this at different temperatures. On incubation at 37°C the LPL activity remained essentially stable for one hour. At higher temperatures the lipase became unstable. Based on these results we decided to use 45°C for gentle inactivation of LPL aimed to prevent aggregation of the enzyme. Chromatography on heparin-Sepharose of active and inactivated LPL showed, as expected [19], that the active form of LPL eluted around 1 M NaCl, while the inactive form(s) eluted earlier in the salt gradient. After 30 min at 45°C most of the lipase eluted early in the gradient. Only about 20 % remained in the form with high heparin affinity. At 60 min this form had been reduced to 8 % and after 90 min it had virtually disappeared. From this we decided to use incubation at 45°C for 90 min to transform LPL to the inactive (presumably monomeric) form with low affinity for heparin. A trace amount of ^125^I-labeled LPL was included in the preparation, to enable us to follow the distribution and metabolism of the injected material. Each rat received about 40 μg lipase protein, except in the electron microscopy studies where only a trace amount of the labeled lipase was injected.

Antibodies against bovine LPL were raised in a chicken (chicken no 225) and IgG were isolated from egg yolks as previously described [48]. Antibodies against bovine LPL were also raised in a rabbit and IgG were isolated on a Protein A-Sepharose column. Both the chicken and the rabbit antibodies were affinity purified on LPL-Sepharose. They were eluted with 0.2 M glycine at pH 2.7, and 50 mM diethylamine at pH 12, respectively, and immediately dialysed against 10 mM Tris/HCl, pH 7.4. Monoclonal antibody 5D2 to LPL was a kind gift from Dr. J. Brunzell, Seattle. A mouse monoclonal antibody (ED2) against a surface antigen expressed on rat Kupffer cells was obtained from Becton Dickinson, San Diego, CA. Goat anti-rabbit IgG labeled with Alexa Fluor 488, goat anti-chicken IgG labeled with Alexa Fluor 488, and goat anti-mouse IgG labeled with Alexa Fluor 546 were from Molecular Probes, Leiden, The Netherlands. Goat IgG, used for control sections, was from Sigma, St.Louis, MO.

### Preparation of tissue for immunofluorescence studies and confocal microscopy

Small pieces of liver were mounted in Tissue – Tec OCT and snap frozen in propane chilled with liquid nitrogen. The tissue pieces were then stored at -70°C until sectioning. Cryosections were fixed for 10 min in 4 % paraformaldehyde. After rinsing, the sections were blocked in 5 % goat serum for 10 min and then incubated overnight with the primary antibody. All these procedures were made at room temperature. Incubation with the secondary antibody was then for 30 min at 37°C. The sections were rinsed in 0.01 M phosphate 0.15 M NaCl at pH 7.4 and mounted in Vectashield medium (Vector laboratories, Burlingame, CA). The immunostained samples were analyzed by confocal laser scanning microscopy (Leica SP2 or Nikon Eclipse E 800). To avoid potential signal crossover the two fluorophores were sequentially scanned. Data were collected with sequential laser excitation to eliminate bleed through and with confocal parameters such as pinhole size set to minimize the thickness of the optical sections. The images were digitally optimized using the Adobe Photoshop software.

### Electron microscopy

For autoradiographic studies, ^125^I-labeled LPL was injected to rats and 10 min later, the livers were perfused with 2% paraformaldehyde, 2.5% glutaraldehyde in 0.1 M phosphate buffer at pH 7.4 for 10 min. After fixation the livers were washed in 0.1 M phosphate buffer, cut in small pieces, dehydrated through graded acetone solutions and embedded in Spurr resin. Postfixation with 1% osmium tetroxide was not performed due to its known effect of fading latent images in autoradiography. Ultrathin sections, 70 nm thick, were collected over Formvar-carbonated copper grids. These sections were coated with a monolayer of Ilford L4 nuclear emulsion, diluted 1:4 with distilled water, by means of a tungsten wire loop following the "loop interference" technique. After an exposure of 8 months, the silver grains were revealed with Phenidon development. Sections were stained with uranyl acetate and lead citrate and examined in a Hitachi MT 800 electron microscope at 75 kV. Quantitative analysis was performed by counting the number of silver grains per area in each experimental condition. The area analyzed was about 25 × 10^4 ^μm^2^.

### Lipase assays

The activities of LPL and of hepatic lipase were determined as described [49]. In the assay for LPL the substrate was an emulsion of soybean triglycerides and a trace amount ^3^H oleic acid-labeled triolein in egg yolk phospholipids. Hepatic lipase (HL) was inhibited by incubation of the samples on ice for two h with rabbit anti-HL IgG. In the HL assay, LPL is inactivated by 1 M NaCl. Both assays were run at 25°C for 30 min. All determinations were carried out in triplicate. The activities are expressed in mU/ml plasma. One mU corresponds to 1 nmol of fatty acid released / min. All determinations were carried out in triplicates.

Plasma samples were stored frozen at -70°C before the analysis. Tissue samples were rinsed in cold 0.9 % NaCl, blotted dry, weighed and then immediately frozen in liquid nitrogen in 9 volumes of buffer at pH 8.2 containing per ml: 1 mg BSA, 10 mg Triton X-100, 1 mg SDS, 5 IE heparin, and protease inhibitor Complete Mini (Roche) 1 tablet / 50 ml buffer. They were stored at -70°C and later thawed and homogenized with a Polytron homogenizer (PT-MR 3000; Kinematica AG, Littau, Switzerland). The homogenates were centrifuged for 15 min at 3000 rpm in a Beckman Microfuge and the supernatants were used for the assays. For assay of LPL in liver and post-heparin plasma, the activity of hepatic lipase was suppressed by incubating the extract with an excess of anti-HL immunoglobulins before assay.

Detergent containing extraction buffers are needed to solubilize and stabilize active and inactive forms of LPL efficiently [19,50], but the detergents may interfere with the assay. Bergö and Olivecrona [19] used the same assay conditions as in the present study and found that the assay system tolerated at least 10 μl of the detergent buffer without any decrease in LPL action. We have repeated these studies in the context of the present experiments. With extracts from adipose tissue, the assay system showed good linearity between the amount of extract added and the lipase activity displayed, but with extracts from heart, kidney or liver there was a definite nonlinearity. Our interpretation is that other tissue proteins, solubilized by the detergents, interfere. We have therefore used a small volume of tissue extract (usually 2 μL), to stay within, or close to, the linear range of the assay. To explore the recovery of LPL activity in liver extracts as prepared here, we added purified bovine LPL to the homogenate which was then treated and assayed as the other samples. The recovery of the added bovine LPL was complete within experimental error.

LPL protein mass was measured by an ELISA, using chicken antibodies for capture and the monoclonal 5D2 antibody coupled to peroxidase for detection [19]. Bovine LPL was used as standard.

## List of abbreviations

LRP – low density lipoprotein receptor-related protein, LPL – lipoprotein lipase, HSPG – heparan sulphate proteoglycan, HL – hepatic lipase, ELISA – enzyme-linked immunoassay, BSA – bovine serum albumin, SDS – sodium dodecyl sulphate, VLDL – very low density lipoprotein

## Authors' contributions

LN carried out the immunolocalization studies, C L-I carried out the electron microscopic studies, SV participated in the design of the study and supervised the electron microscopy, JG carried out the studies on wash-out of LPL from tissues after heparin, TO conceived of the study, participated in its design and drafted the manuscript. GO participated in the design of the study and coordinated the work. All authors read and approved the final manuscript.

## Figures and Tables

**Table 1:** Lipases in plasma and liver after injection of heparin

*Time min*	*Plasma*									*Liver*											
	
	LPL									LPL									HL		
	
	Activity mU/ml			mass ng/ml			spec act mU/ng			activity mU/ml			mass ng/ml			spec act mU/ng			activity mU/g		
0	8	±	2	29	±	1	0.28	±	0.06	26	±	1	119	±	12	0.22	±	0.02	450	±	21
2	844	±	35	746	±	62	1.17	±	0.11	116	±	21	243	±	19	0.46	±	0.04	181	±	22
15	1161	±	146	1047	±	190	1.18	±	0.11	245	±	30	397	±	37	0.62	±	0.05	181	±	23
60	485	±	124	502	±	118	1.24	±	0.15	265	±	27	624	±	66	0.45	±	0.06	255	±	17

Rats were injected with heparin. After the indicated times the rats were killed, blood samples were taken from the heart and livers were removed and processed for determination of LPL activity and mass as described in Material and Methods. The values have been corrected for the contribution of blood remaining in the liver, and are means ± SEM, n = 6.

**Table 2:** Effect of heparin injection on tissue LPL activities

*Treatment*	*LPL activity mU/g*				
	
	Plasma	Liver	Heart	Epididymal adipose	Perirenal adipose
Fed rats					
Before heparin	1.9 ± 0.2	28 ± 8	999 ± 111	1700 ± 138	1094 ± 195
After heparin	1057 ± 59^a^	173 ± 20^a^	868 ± 67	938 ± 70^a^	383 ± 73^a^
Fasted rats					
Before heparin	2.5 ± 0.3	54 ± 12	1199 ± 63	345 ± 48^b^	188 ± 49^b^
After heparin	374 ± 76^a,b^	189 ± 21^a^	729 ± 70^a^	357 ± 56^b^	263 ± 28

The fasted rats had been deprived of food overnight (16 – 18 hours). The values have been corrected for the contribution of blood remaining in the liver, and are means ± SEM, n = 5. The difference is statistically significant (p < 0.05) ^a ^when comparing the effect of heparin,^b^when comparing the effect of nutritional state.

**Table 3:** Clearance of ^125^I-labeled LPL from blood and uptake in liverin the presence and absence of heparin

*Time after injection (min)*	*% of injected ^125^I-labeled LPL*		
	
	No heparin		Heparin
	Blood	Liver	Liver

Active LPL			
2		32.6 ± 3.1	13.9 ± 0.6
15	8.5 ± 13.6	51.5 ± 2.8	30.4 ± 1.4
60	6.7 ± 5.5	18.2 ± 1.7	22.8 ± 0.4
Inactive LPL			
15	9.4 ± 0.7	50.0 ± 5.5	
60	6.2 ± 1.0	18.8 ± 3.1	

Rats were injected with active or inactive ^125^I-labeled LPL either directly, or five min after intravenous injection of heparin. After the indicated times the rats were killed, blood samples were taken from the heart and livers were removed. The values have been corrected for the contribution of blood remaining in the liver, and are means ± SEM, n = 5.
